# Luteolin inhibits A549 cells proliferation and migration by down-regulating androgen receptors

**DOI:** 10.1186/s40001-023-01302-4

**Published:** 2023-09-16

**Authors:** Xu Li, Yeling Tang, Pengchen Liang, Miaomiao Sun, Tian Li, Zhiping Shen, Shuang Sha

**Affiliations:** 1 Tongji University School of Medicine, Shanghai, 200092 China; 2 General practice, Tongji University School of Medicine Affiliated Anting Community Health Center of Jiading District, Shanghai, 201805 China; 3 Graduate School, Shanghai University of Traditional Chinese Medicine, Shanghai, 200120 China; 4 School of Microelectronics, Shanghai University, Shanghai, 201800 China; 5 Graduate School, China Pharmaceutical University, Nanjing, 211198 China; 6 Shanghai Key Laboratory of Molecular Imaging, Shanghai University of Medicine and Health Sciences, Shanghai, 201318 China

**Keywords:** Yi Fei Qing Hua Granules, Luteolin, A549 cancer cells, Androgen receptor

## Abstract

**Background:**

Yi Fei Qing Hua Granules (YQG) is a traditional Chinese herbal medicine with the effects of inhibiting the proliferation of lung cancer cells. Luteolin is one of the active compounds of YQG. Luteolin is a common flavonoid extracted from natural herbs and it can promote cancer cells apoptosis has been reported. However, the underlying molecular mechanism and effects of luteolin on human lung cancer needs to be validated.

**Methods:**

Molecular docking, network pharmacology methods and quantitative structure–activity relationship (QSAR) model were used to identify the active components of YQG and their possible mechanisms of action. Western blot analysis was used to measure AR expression in A549 cells. Cell migration assays were used to detect A549 cells proliferation transfected by AR plasmid and AR mutation plasmid, respectively.

**Results:**

TCMSP search results revealed that there are 182 active compounds in YQG, which correspond to 232 target genes. Sixty-one genes were overlapping genes in the 2 datasets of TCMSP and GeneCards. Through bioinformatics tagging of these overlapping genes, a total of 1,951 GO functional tagging analysis and 133 KEGG pathways were obtained. Through molecular docking technology and QSAR model verification, the multi-target active compound luteolin was screened out as one of the active components of YQG for in vitro verification. Androgen receptor (AR) was the hub protein with the highest docking score of luteolin. Western blot showed that luteolin could inhibit AR protein expression in lung cancer cell line A549. After the phosphorylation site of AR protein 877 was inactivated, the ability of luteolin to inhibit the proliferation of lung cancer cells was weakened. Luteolin significantly inhibited the growth of A549 xenogeneic tumors at day 25 and 28 and inhibited the expression of AR.

**Conclusion:**

In this study, we have explored luteolin as one of the active components of YQG, and may inhibit the proliferation and migration of A549 cells by decreasing the expression of AR and the regulation of phosphorylation at AR-binding sites.

## Background

Lung cancer is the leading cause of cancer death worldwide and is highly prevalent in China [1]. Most patients are diagnosed with non-small-cell lung cancer (NSCLC) and have a 5-year survival rate of only 18% [2]. At present, in China, multi-disciplinary comprehensive treatment is the main treatment option for lung cancer, including surgery, radiotherapy and chemotherapy, molecular targeted therapy, immunotherapy, and traditional Chinese medicine (TCM) [3, 4]. TCM is one of multi-target strategies for cancer therapeutics based on its whole system treatment [5], and it has unique advantages in the treatment of NSCLC. In addition to its direct anti-tumor effect, TCM can also be combined with radiotherapy and chemotherapy to enhance the therapeutic efficacy, reduce toxicity, and limit drug resistance [6, 7]. Previous studies have shown that compared with chemotherapy alone, adjuvant chemotherapy (vinorelbine plus cisplatin (DDP) or vinorelbine plus carboplatin, NP/NC) together with TCM treatment after NSCLC resection can improve the quality of life of patients, and TCM can also enable patients to complete adjuvant chemotherapy more safely and effectively [8, 9].

One of the Chinese medicines for the clinical treatment of lung cancer, Yi Fei Qing Hua granules (YQG), has such an effect. YQG is mainly composed of Astragalus, Codonopsis, Glehnia littoralis, Ophiopogon, Agrimonia, Fistula, Patriniaceae, Oldenlandia diffusa, Fritillaria, Aster, Platycodon, bitter almond, and licorice. Pharmacological studies have found that YQG demonstrates anti-cancer, immunoregulation, and tumor angiogenesis–inhibitory effects, and clinical observations and experimental studies have shown that its prescription has a certain effect on the treatment of NSCLC [10]. However, the exact mechanism of action of YQG is not yet clear.

Androgen Receptor (AR) is a ligand-dependent transcription factor which belongs to the steroid hormone nuclear receptors superfamily. AR is up-regulation in the prostate cancers, and is a valuable therapeutic target in prostate cancer [11, 12]. Studies have shown that a variety of cytokines, kinases and downstream signaling factors promote the growth of tumor cells through the interaction of AR or androgen-independent pathway [13, 14]. In the low hormone environment, other alternative pathways, including MAPK pathway, Wnt/β-catenin and c-Myc, also play an important role in promoting the transformation of castration-resistant prostate cancer (CRPC) [15, 16]. The interaction of AR with androgens is accompanied by physiological functions and structural changes of AR, such as phosphorylation [17]. However, the phosphorylation of androgen receptor is independent of hormones. For instance, EGFR and its ligands, could not only take the place of androgens to promote androgen receptor phosphorylation but also serve as co-regulators of androgen receptors to enhance the activation of downstream target genes [18].

Whether NSCLC is a sex hormone-related tumor is still controversial, the cancer genome atlas (TCGA) has identified androgen receptor (AR) to be mutated, deleted and amplified across human lung squamous cell carcinoma. Expression of AR is critical for early lung development [19].

In the present study, we found that lung cancer A549 cells could express androgen receptor (AR). We used network pharmacology, computer molecular docking, and bioinformatics technology to study the effective ingredients and mechanism of Yi Fei Qing Hua Granules (YQG) inhibited lung cancer AR expression and proliferation. This study was validated in combination with in vitro experiments to provide a scientific basis for the mechanism of the anti-cancer effect of YQG.

## Materials and methods

### Target database construction

Lung cancer-related genes were collected from GeneCards. The chemical constituents and target genes of 13 Chinese herbal medicines in YQG were screened using the TCM System Pharmacology Database (TCMSP) (https://tcmsp-e.com/). The screening conditions were oral bioavailability (OB) of at least 30% and drug-like (DL) degree of at least 0.18. In addition, UniProt was used to verify the gene symbols of the genes screened in TCMSP and to construct a dataset of Chinese medicine chemical components and targets.

### Data cleaning and drawing

R software (version 3.6.3) was used to calculate the overlapping genes between lung cancer-related genes and known YQG lung cancer treatment target genes to construct a protein–protein interaction (PPI) network. The STRING database (version 11.0) was then used to analyze the interaction of target genes in the network. To ensure the robustness of the results, when the interaction score was greater than or equal to 0.4, the network type was selected as a physical subnetwork, and the Cytoscape software program (version 3.7.1) was used for analysis. The top ten proteins with three-dimensional structure with the strongest interaction between proteins were selected as the main target proteins (hub proteins).

### Molecular docking

We downloaded the corresponding three-dimensional structure diagram of the active compounds and the proteins related to the hub genes from PubChem and the Protein Data Bank, respectively, and then used Discovery Studio (DS) 2019 software program (Dassault Systèmes, Vélizy-Villacoublay, France) for molecular docking and absorption, distribution, metabolism, and excretion (ADME) prediction.

### Quantitative structure activity relationship (QSAR)

Using the method of machine learning, the hub proteins and the top 10 multi-target active components screened were analyzed based on QSAR. Download data: Use the ChEMBL (https://www.ebi.ac.uk/chembl/) database to obtain the biological activity data of drug molecules that act on hub proteins as a training data set for modeling. The SMILES sequences of the top 10 multi-target active ingredients were obtained from the PubChem (https://pubchem.ncbi.nlm.nih.gov/) database. Descriptor calculation and feature selection: Use the PaDEL-Descriptor library in python to calculate the molecular descriptors of compounds. The input to the PaDEL-Descriptor software is the SMILES sequence of the molecule, and then features with low variance are removed using the python sklearn library feature selection method Variance Threshold. Model selection: Use the LazyRegressor function in python’s lazypredict library for automatic training, compare 41 machine learning models, and use the Adjusted R-squared, R-squared, and root mean square error (RMSE), time taken four criteria to evaluate the effect of the model, select the model with the best evaluation results to establish the QSAR model of the effective Chinese medicine monomer in the treatment of lung cancer. Use the four indicators of explained_variance_score, mean_absolute_error (MAE), mean_squared_error (MSE), and R-squared value (r2_score) provided by the sklearn library metrics to judge the reliability of the regression model. Finally, the descriptors of TCM monomer molecules are input into the model to predict the biological activity value.

### Cell culture

The human lung cancer cell lines PC9, A549, and H460 were provided by the Clinical Research Center of Jiading District Central Hospital. They were cultured in Dulbecco’s Modified Eagle Medium (DMEM) (Gibco Fisher Scientific, Beijing, China) containing 10% fetal bovine serum (Gibco) at 37 °C, with 5% CO_2_ and 95% air humidity, plus 100 U/mL of penicillin and 100 U/mL of streptomycin (Gibco).

### Cell proliferation test

The cell counting kit 8 (Dongren Chemical Technology Co., Ltd., Japan) colorimetric method was used to detect the effect of luteolin (Chengdu Herbpurify Co., Ltd., Chengdu, China) on the proliferation of A549, PC9, and H460 cells and the effect of luteolin on A549 cells after mutation of androgen receptor phosphorylation sites at the binding site. The cells were inoculated in a 96-well plate (1 × 10^4^ cells/well), incubated alone at 37 °C for 24 h, then incubated with different doses of luteolin in a 5% CO_2_ incubator at 37 °C for 48 h. Luteolin (80 µM) was applied to A549 cells after transfection of AR wild-type and mutant-type plasmids for 24 h. Next, 10 µL of CCK-8 was added to each well, followed by incubation for one hour. The optical density value was measured at 450 nm using a microplate reader (Thermo Fisher Scientific, MA, USA). Each experiment was repeated three times.

### Cell scratch test

A549 cells were inoculated in a six-well plate (3 × 10^5^ cells/well), and a scratch test was performed after 24 h of attachment, where we added 80 μM luteolin and took pictures of scratches at 0, 24, and 48 h, respectively. Each experiment was repeated three times. Luteolin (80 μM) was applied to untransfected A549 cells (Control), A549 cells transfected with empty vector plasmids (NC), AR wild-type (AR-WT) and mutant-type plasmids (AR-MU) for 24 h, and scratch pictures were taken at 0, 24, and 48 h. The cell migration rate was calculated using Image J (version 1.52a; National Institutes of Health, Bethesda, MD, USA), and the GraphPad Prism software program was used for statistics and graphing.

### Lactate dehydrogenase assay

A lactate dehydrogenase (LDH) cytotoxicity detection kit was purchased from Beyotime Biotechnology (Shanghai, China). The 2 × 10^3^ A549 cells per well were inoculated in a 96-well plate. After 24 h, the cells adhered to the wall and the medium was removed. Cells were washed once with PBS and serum-free medium containing different concentrations of luteolin was added to the cells for 47 h. Then, LDH release reagent was added, everything was mixed by pipetting several times, and further incubation was completed in the presence of luteolin effect lasting for 48 h. Next, 120 μL of the supernatant was collected from each well and added to a new 96-well plate, and the sample was measured at an optical density of 490 nm.

### Flow cytometry (FCM) analysis of apoptosis

A549 cells were seeded on a 6-well culture plate for 12 h, treated with different concentrations of luteolin for 48 h and collected. The cells were resuspended with PBS (4 °C) and centrifuged at 2000 rpm for 5–10 min more, and then washed. According to the instructions for the production of the Annexin V-fluorescein isothiocyanate/propidium iodide (Annexin V‐FITC/PI) Apoptosis Kit (BD Biosciences, CA, USA), the cells were suspended with 500 μL of binding buffer, and 5 μL of Annexin V-FITC and PI were added and incubated at room temperature and in the dark for 15 min. Cell apoptosis was detected by flow cytometer (BD FACS Canto II).

### Construction of AR wild-type plasmid and AR mutated plasmid

AR wild-type plasmid was constructed by primers F and R to amplify the target gene. The primer sequences are listed in Table 1. PCR amplification conditions were as follows: 66 °C annealing temperature, 68 °C extension temperature for 1 min and 30 cycles. The DNA fragment was collected and digested by the EocR I and BamH I (Takara Bio, Shiga, Japan) simultaneously. The vector pLenO-GTP-C-3X flag (Zorin Biological Technology Co., Ltd., Shanghai, China) was digested with the same condition and ligated with the target fragment by Fusion DNA ligase.

**Table 1:** The PCR primer sequences

Name	Sequences
AR-F	TAGAGCTAGCGAATTCATGGAAGTGCAGTTAGGGCTGG
AR-R	CTTTGTAGTCGGATCCCTGGGTGTGGAAATAGATGGGC
AR-MUT-F	GAGAGAGCTGCATCAGTTCGCATTTGACCTGCTAATCAAGT
AR-MUT-R	ACTTGATTAGCAGGTCAAATGCGAACTGATGCAGCTCTCTC

AR mutant expression plasmids (AR-MU) were constructed using the point mutation PCR method. Using primers F and R-MUT to amplify the region fragments, and primers F-MUT and R to amplify the region fragments, both DNA fragments were mixed and used as templates for PCR amplification, and primers F and R are used to amplify the fragments of the target gene. The PCR amplification conditions were the same as with the AR wild-type plasmid.

### Transfection of AR plasmids

A549 cells were inoculated in a six-well plate (3.5 × 10^5^ cells/well). Then, we mixed the empty vector, wild-type (WT) AR, and the mutant AR plasmids with lipofectamine 2000 (Thermo Fisher Scientific) at a ratio of 5 μg to 8 μL, and transferred them into different wells of the six-well plate. Next, 2 mL of serum-free and dual-antibody-free DMEM was added to each well. After 4 h, the medium with serum and non-double antibody was replaced. Twenty-four hours after transfection, transfection was observed under a fluorescence microscope (Leica Camera, Wetzlar, Germany).

### Western blot

A549 (7 × 10^5^/well) cells were seeded in a six-well plate overnight. After the cells adhered, DMEM complete medium containing luteolin 40 μM or 80 μM was added to three wells of the six-well plate, and treated for 48 h. Cells were collected after rinsing and scraping with PBS. Cells were lysed on ice with lysis buffer (Beyotime Institute of Biotechnology, Shanghai, China). The concentration of protein was quantified using a BCA kit (Tiangen Biotech, Beijing, China). An equivalent amount of protein was loaded on 10% SDS-PAGE gels and then transferred to nitrocellulose membranes. The membranes were blocked with 5% non-fat milk for an hour, and then incubated with primary antibodies (1:1000) at 4 ℃ overnight. The antibodies were as follows: GAPDH (YOBIBIO, Shanghai, China), AR (Cell Signaling Technology, MA, USA). On the next day, the membranes were washed three times (5 min each time) with TBST solution, and incubated with secondary antibody (1:5000) for 2 h. After three washes with TBST (10 min each time), images were acquired using a gel imaging system (Thermo Fisher Scientific, Waltham, MA, USA).

### Animal experiments

All animal studies were approved by the Ethics Committee of Shanghai University of Medicine and Health Sciences (Shanghai, China). Male Balb/c nude mice 6–7 weeks of age were purchased from Daoke Medical Technology Co., LTD (Shanghai, China). 1 × 10^7^ A549 cells in PBS were injected subcutaneously to the right side of the back of each nude mouse. When the tumor volume reached ∼84 mm^3^, the mice were randomly divided into four groups according tumor volume (ten mice per group). In the control group, 10 μl/g 10% DMSO/corn oil (vehicle) was given through intragastric administration. Mice in the experimental groups were given 150 mg/kg luteolin oral gavage, 1 mg/kg 5α-Dihydrotestosterone (DHT, Selleck, USA) (0.2 mg/ml) subcutaneous injection, and 150 mg/kg luteolin combined with 1 mg/kg DHT subcutaneous injection). All treatments were observed every 2 days for 28 days. The weight of the experimental animals was measured about once a week after inoculation until before grouping, and the tumor volume was measured once a week when the tumor was visible. After grouping, the weight of experimental animals and the tumor volume of experimental animals were measured twice a week. The mice were euthanized 1 days after the last dose and tumors were removed and photographed. Tumors were fixed in 4% paraformaldehyde for 12 h, embedded in paraffin, and cut into 5-μm sections. The sections were assigned for hematoxylin–eosin (HE) stain and immunohistochemistry (IHC) assay and examined by light microscopy. For IHC, anti-AR (1:500; Cell Signaling Technology, MA, USA) antibodies were used.

### Statistical analysis

All of the experiments were repeated three times. Results are expressed as mean and standard deviation (SD) values, and all of the statistical comparisons were evaluated using one-way or two-way analysis of variance. Significant differences between means were measured using Duncan’s multiple range test. Statistical analysis was performed using GraphPad Prism software (version 8.0.2) and R-studio (R version 3.6.3). *P* < *0.05*indicated that the difference was statistically significant.

## Results

### Screening of active ingredients

**Fig. 1:**
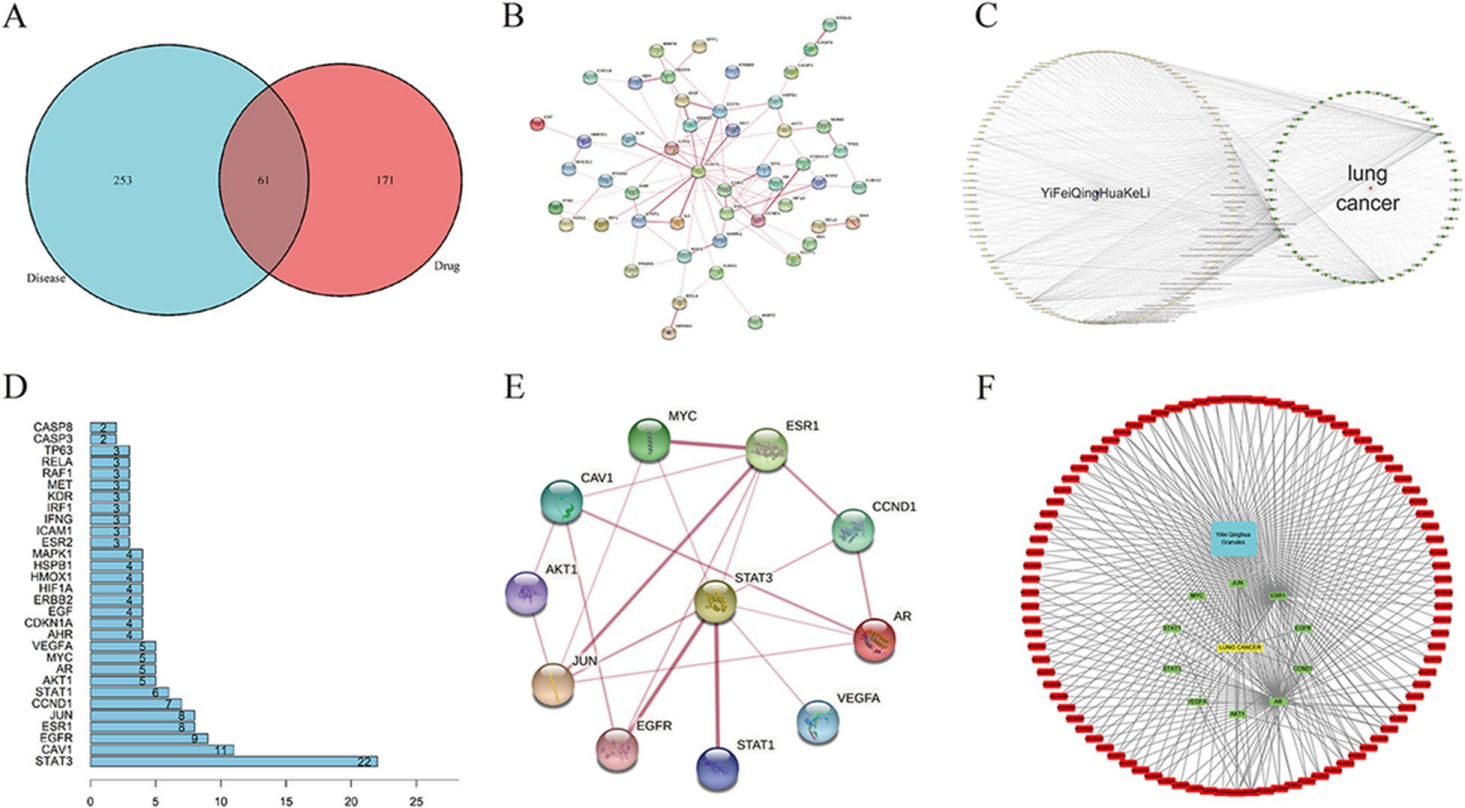
Screening of active ingredients in YQG. **A** The 61 interactive genes that overlap the possible targets of YQG and the lung cancer gene dataset. **B** The interaction network of 61 proteins was constructed using STRING (version 11.0). **C** The relationship between 61 interactive genes and YQG active ingredients. **D** Ranking of the genes with the strongest protein interactions. **E** PPI network of interactions between 10 hub genes. **F** 10 hub genes represent proteins associated with the active components of YQG

182 compounds of 13 Chinese herbal medicines were identified as “qualified” by a database search and literature search. A total of 232 genes were predicted as possible targets for YQG. Meanwhile, 314 lung cancer genes with lung cancer-related gene scores above 40 points were screened by Gene Cards to create a lung cancer gene dataset. In total, there were 61 interacting genes that overlapped with each other (Fig. 1A), and an interaction network of 61 proteins was constructed by using STRING version 11.0 (Fig. 1B). The interaction diagram of these 61 interacting genes with YQG active components was drawn by using the Cytoscape and R software programs (Fig. 1C). The ten genes with the strongest protein interaction associations (*STAT3*, *EGFR*, *ESR1*, *JUN*, *CCND1*, *STAT1*, *AKT1*, *AR*, *MYC*, and *VEGFA*) were selected as hub genes. Since *CAV1* has no three-dimensional protein structure, DS was unable to conduct molecular docking, so it was temporarily excluded (Fig. 1D). In order to explore the interaction between the ten YQG-related hub genes, another PPI network was constructed (Fig. 1E), and the ten hub gene expression proteins were associated with the active components related to YQG (Fig. 1F). In this network, the red outer circle seen in Fig. 1F represents the compound, the green represents the proteins corresponding to the 10 hub genes, the yellow represents the disease, and the blue represents the drug. An ADME prediction of the 91 hub gene-related compounds was performed using DS. Following the above screening, we found that quercetin, luteolin, kaempferol could dock with six, five and four proteins expressed by hub genes, respectively (Table 2), and are multi-target active compounds. Meanwhile, ADME predicted that luteolin had better oral absorption and utilization efficiency. Bioactivity values predicted by QSAR model (Table 3) showed that luteolin had higher bioactivity values among the top ten multi-target compounds. Therefore, luteolin was selected as the active compound for subsequent experimental verification. Combined with the results of molecular docking, QSAR model validation and literature review, we chose luteolin as the active compound for the next step of molecular biology experiment validation.

**Table 2:** Molecular docking results

Molecule name	Pubmed CID	Protein	Score
Quercetin(MOL000098)	5280343	AR	113.553
	5280343	AKT1	110.056
	5280343	MYC	108.57
	5280343	CCND1	93.3471
	5280343	EGFR	92.4824
	5280343	STAT1	87.9574
Luteolin(MOL000006)	5280445	AR	114.675
	5280445	AKT1	110.1
	5280445	EGFR	101.892
	5280445	CCND1	95.1228
	5280445	VEGFA	94.9252
Kaempferol(MOL000422)	5280863	AKT1	112.736
	5280863	AR	109.408
	5280863	STAT1	87.1065
	5280863	JUN	40.3892
Naringenin(MOL004328)	439246	AKT1	112.636
	439246	ESR1	101.766

**Table 3:** Predicted bioactivity values of the top ten multi-target active compounds

Molecule name	STAT3	STAT1	AKT1	MYC	AR	SUM	Number of targets
Quercetin	4.042564	4.376751	6.823909	3.779892	6	25.02312	6
Luteolin	4.649752	4.376751	6.823909	3.779892	5	24.6303	5
Kaempferol	4.885389	4.376751	4.263763	3.779892	4.849485	22.15528	4
Licochalcone A	4.522879	4.376751	8	3.779892	5	25.67952	3
Naringenin	4.042564	4.376751	9.420216	3.779892	4.849485	26.46891	2
7-Acetoxy-2-methylisoflavone	4.30103	3.395774	5	5.592397	6	24.2892	2
Glabridin	4.042564	4.376751	6.451893	3.779892	5	23.6511	2
Phaseolinisoflavan	4.042564	4.730487	5	3.779892	5.173394	22.72634	2
Licoisoflavanone	4.042564	4.376751	5	3.779892	4.849485	22.04869	2
7-Methoxy-2-methyl isoflavone	4.042564	4.730487	4.263763	3.779892	4.849485	21.66619	2

### Luteolin inhibits the proliferation of lung cancer cells

**Fig. 2:**
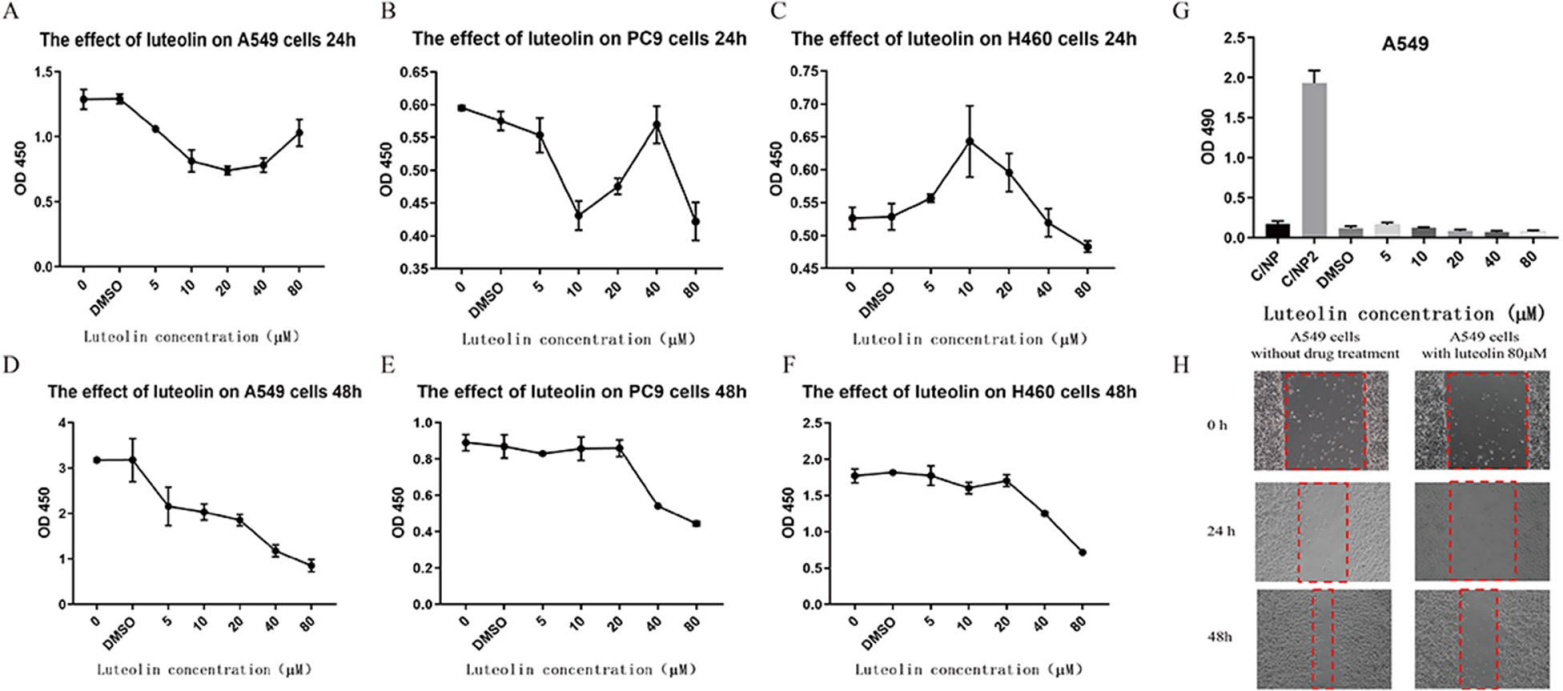
Cellular viability and toxicity were detected by CCK8 and LDH assays. **A**–**C** The proliferation of A549, PC9, and H460 cells was inhibited by different concentrations of luteolin for 24 h. **D**–**F** The proliferation of A549, PC9, and H460 cells was inhibited by different concentrations of luteolin for 48 h. **G** Toxicity damage of luteolin to A549 cells was measured by the release of LDH. **H** Wound healing assay of A549 cells treated with 80 μM luteolin

A CCK-8 assay was performed to examine the inhibition of A549, PC9, and H460 cell proliferation by luteolin (0 μM, 5 μM, 10 μM, 20 μM, 40 μM, and 80 μM). As seen from the line chart (Fig. 2A–F), the action of the drug is not stable at 24 h, but the drug level reached an apparent steady state by 48 h. The CCK-8 results suggested that luteolin demonstrated strong inhibitory activity on the proliferation of A549 cells in a dose-dependent manner at 48 h. The concentration of dimethyl sulfoxide (DMSO) was 2.29 μL/mL, which was required for the dissolution of luteolin. As shown in Fig. 2A–F, DMSO had no effect on cell proliferation at this concentration. Among the three lung cancer cell lines, compared with the gefitinib mutant lung cancer cell line PC9 and the large cell lung cancer cell line H460, the wild-type lung adenocarcinoma cell line A549 showed a better concentration dependence on luteolin. As A549 cells have a broader representative significance in lung cancer, follow-up experiments mainly focusing on A549 to screen the docking site and mechanism of luteolin in A549 are warranted.

### Effect of luteolin on the LDH assay

The LDH release experiment was used to detect the effect of luteolin at different concentrations on the lung cancer cell line A549 (because A549 cells have a better concentration dependence on luteolin inhibition of their proliferation), and the results showed that there was no significant difference in the release of luteolin at different concentrations (0 μM, 5 μM, 10 μM, 20 μM, 40 μM, and 80 μM) (Fig. 2G). The results indicated that the toxicity of luteolin to lung cancer cells was not obvious at these concentrations. (C/NP represented the negative control and C/NP2 represented the positive control.)

### Effect of luteolin on A549 cell migration

Cell scratch test was used to observe the migration of A549 cells with 80 μM Luteolin. We observed that after treatment with luteolin at 80 µM, the migration of A549 began to be inhibited after 24 h, and the inhibition effect was more significant at 48 h (Fig. 2H).

### Effect of luteolin on A549 cell apoptosis

**Fig. 3:**
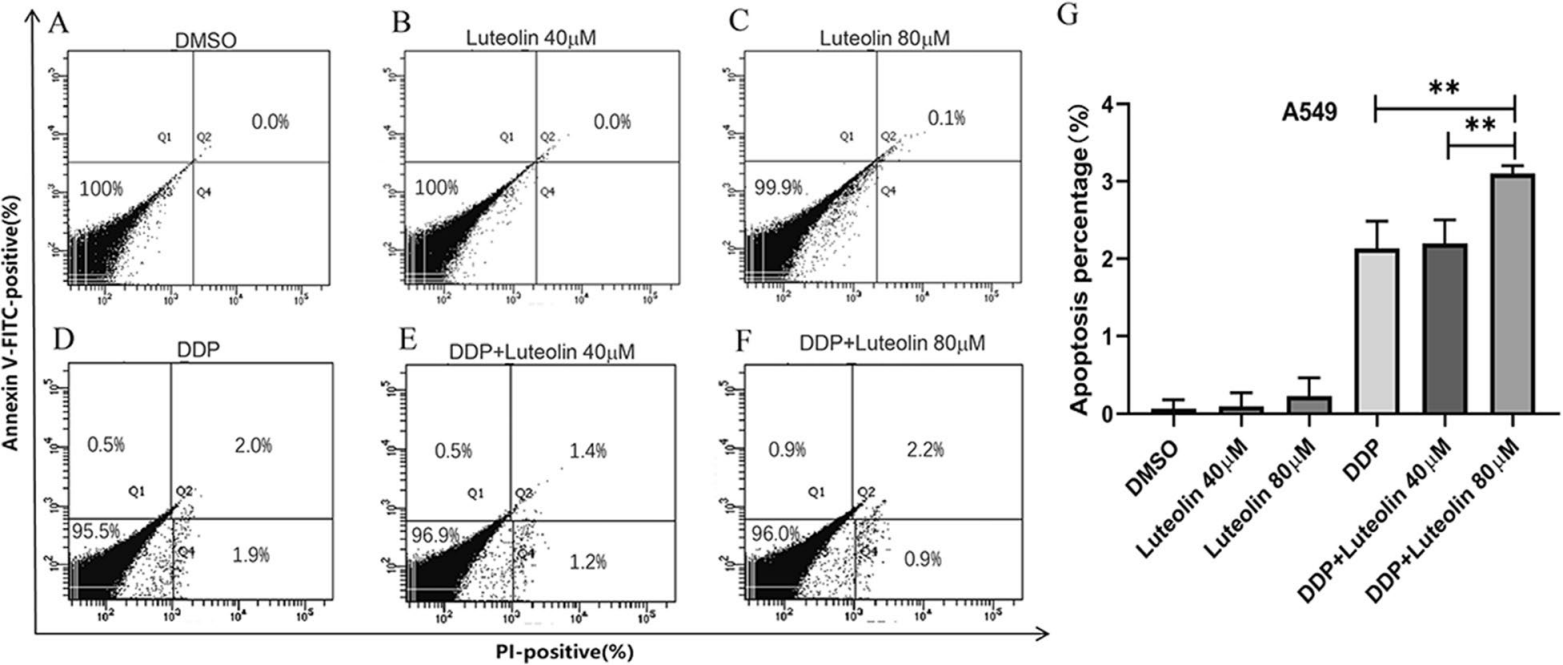
Apoptosis of luteolin on A549 cells. **A** Effect of DMSO (2.29 μL/mL) on apoptosis of A549 cells was detected by FCM. **B**, **C** Effect of 40 μM or 80 μM of luteolin on apoptosis of A549 cells was detected by FCM. **D**–**F** Effect of DDP (10 μg/mL), with 40 μM or 80 μM of luteolin on apoptosis of A549 cells was detected by FCM. **G** Column chart of apoptosis detected by FCM

FCM analysis was used to further verify the pro-apoptotic effect of luteolin on A549 cells. It can be seen from Fig. 3 that, when luteolin was used for 48 h, with concentrations of 40 μM and 80 μM, no obvious apoptosis-inducing effect was seen. When luteolin was used in combination with DDP (10 μg/mL), however, the concentration of 80 μM could induce a significant increase in lung cancer cell apoptosis (**P < 0.01) (vs. 10 μg/mL of DDP alone and 10 μg/mL of DDP with 40 μM of luteolin, respectively).

### Luteolin inhibits cell proliferation by binding to AR phosphorylation sites

**Fig. 4:**
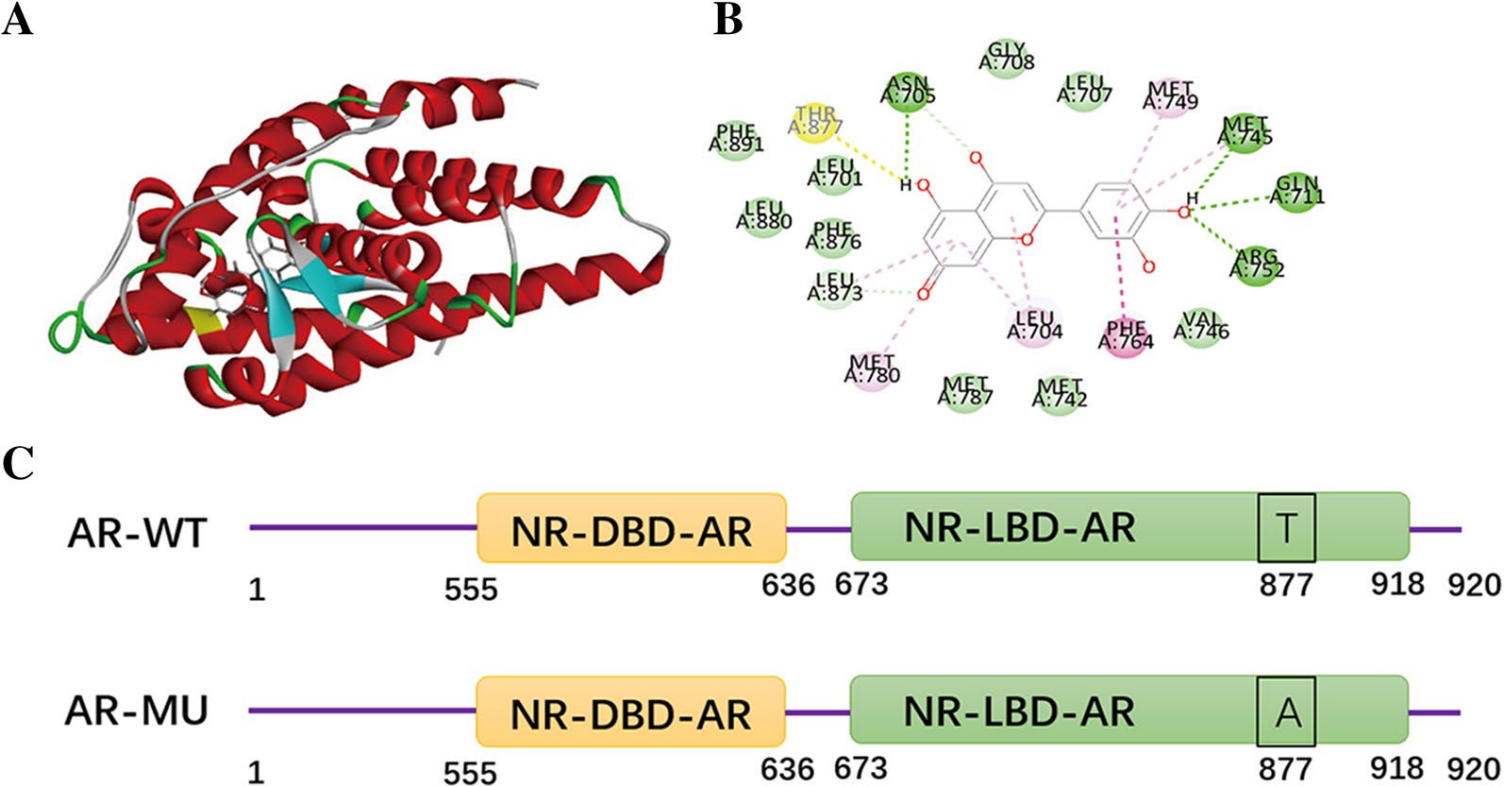
The best bound conformation site of luteolin binding to AR proteins and AR protein mutation sites. **A** The three-dimensional structure diagram of the combination of luteolin and AR protein. **B** Molecular structure plan of luteolin-binding site with AR protein. **C** Schematic diagram of the AR protein mutation site. (*AR-WT* AR wild-type strain, *AR-MU* AR mutant strain, *NR-DBD-AR* DNA-binding domain of AR comprises two C4-type zinc fingers, *NR-LBD-AR* ligand-binding domain of the nuclear receptor androgen receptor, ligand-activated transcription regulator)

**Fig. 5:**
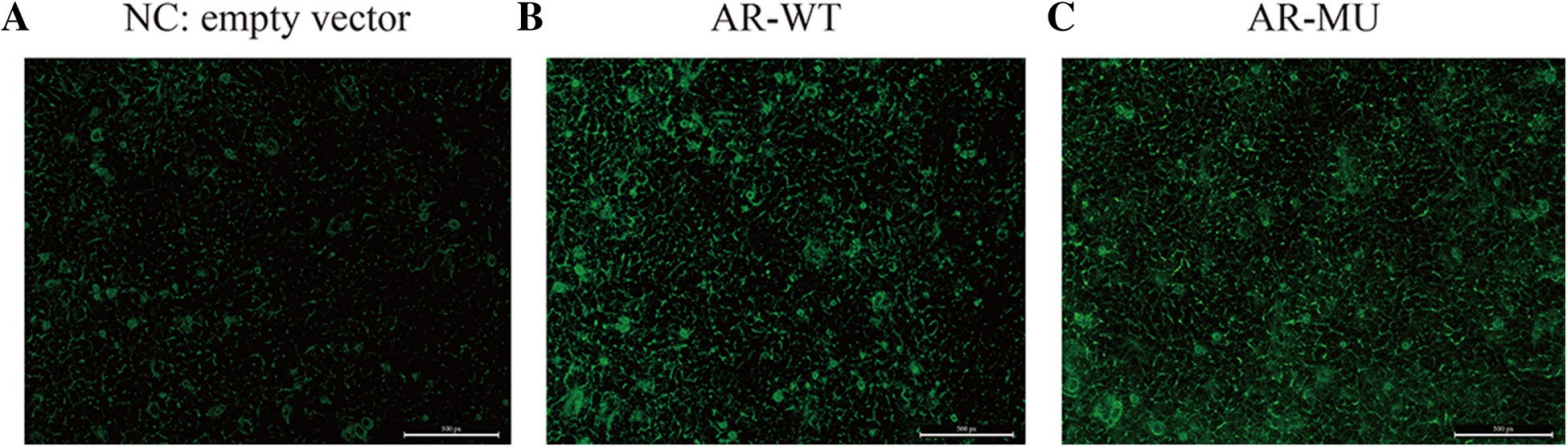
The expression of the plasmid containing green fluorescent protein after transfection of A549 cells. **A** NC: empty vector **B** AR-WT: AR wild-type plasmids **C** AR-MU: AR mutant-type plasmids

**Fig. 6:**
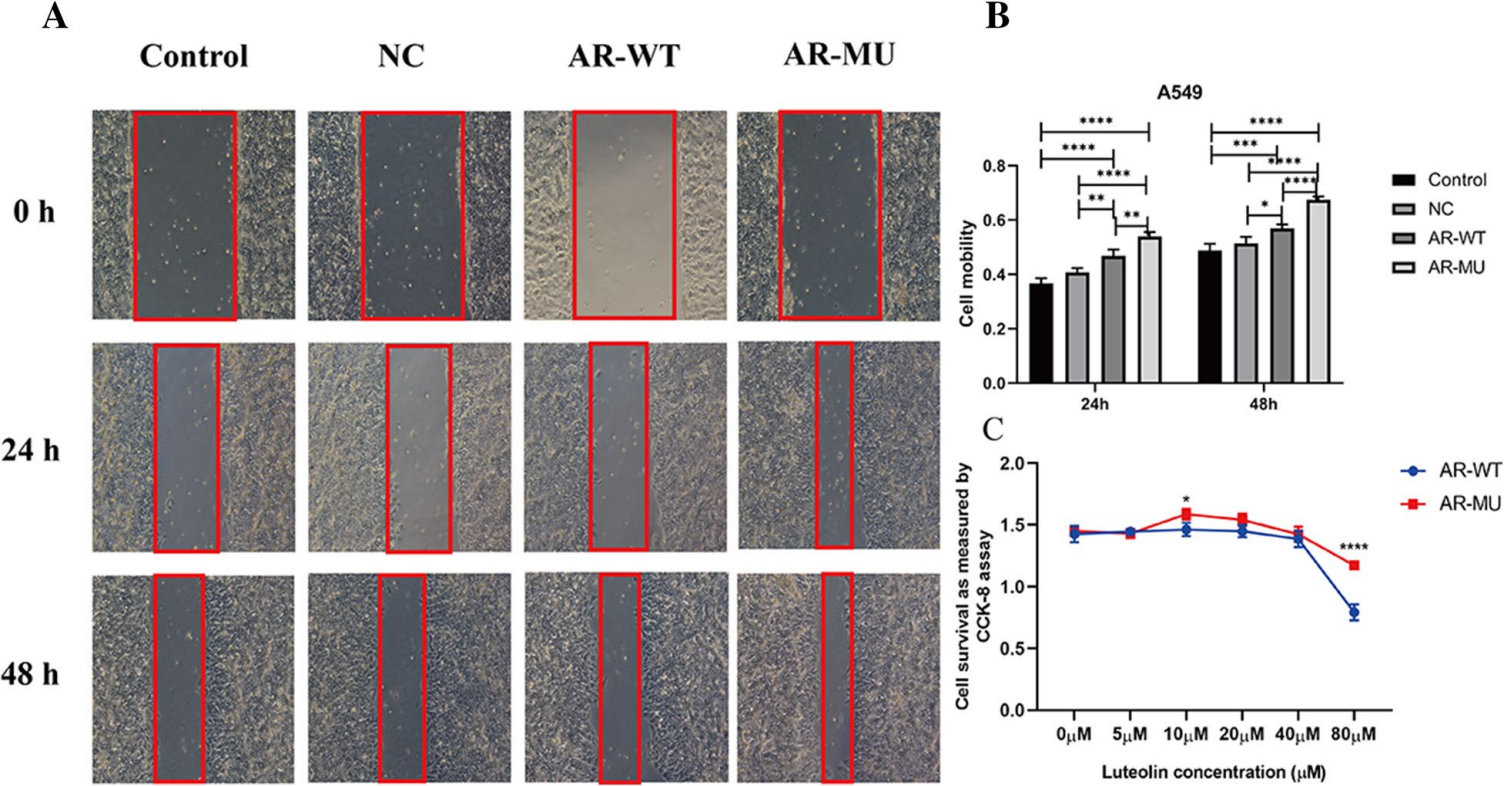
Effects of luteolin on migration and proliferation of transfected cells. **A** Comparison of the effects of 80 μM luteolin on AR sites before and after mutation on the wound-healing ability in the A549 cell line. Control: untransfected A549 cells, NC: A549 cells transfected with empty vector plasmids, AR-WT: A549 cells transfected with AR wild-type, AR-MU: A549 cells transfected with mutant-type plasmids (AR-MU). **B** Column analysis of cell scratch test: *P < 0.05, **P < 0.01, ***P < 0.001, ****P < 0.0001. n = 3. **C** Cell proliferation of wild and mutant strains after treatment with 80 μM of luteolin for 48 h

We used the DS proteomic analysis software to calculate the three-dimensional structure diagram of luteolin and AR binding as well as the sites of their stable interaction (Fig. 4A and B). In this context, THR 877, the binding site of luteolin to AR protein, mutates to ALA, resulting in phosphorylation inactivation (Fig. 4C). A549 cells were transfected with the plasmids containing green fluorescent protein, and the transfection efficiency was observed 24 h later (Fig. 5). At the same time, 80 μM of luteolin administered to A549 cells was transfected with AR wild-type and mutant-type plasmids. The results of scratch images at zero, 24, and 48 h revealed that the inhibitory effect of luteolin on cell migration was weakened following phosphorylation site mutation and inactivation (Fig. 6A and B). Meanwhile, CCK-8 experiment showed that luteolin had a certain inhibitory effect on cell proliferation following the transfection of AR mutation (phosphorylation site inactivation) plasmid, but its inhibitory ability was weaker than that of the control group (Fig. 6C).

### Luteolin inhibited the expression of AR protein in A549

**Fig. 7:**
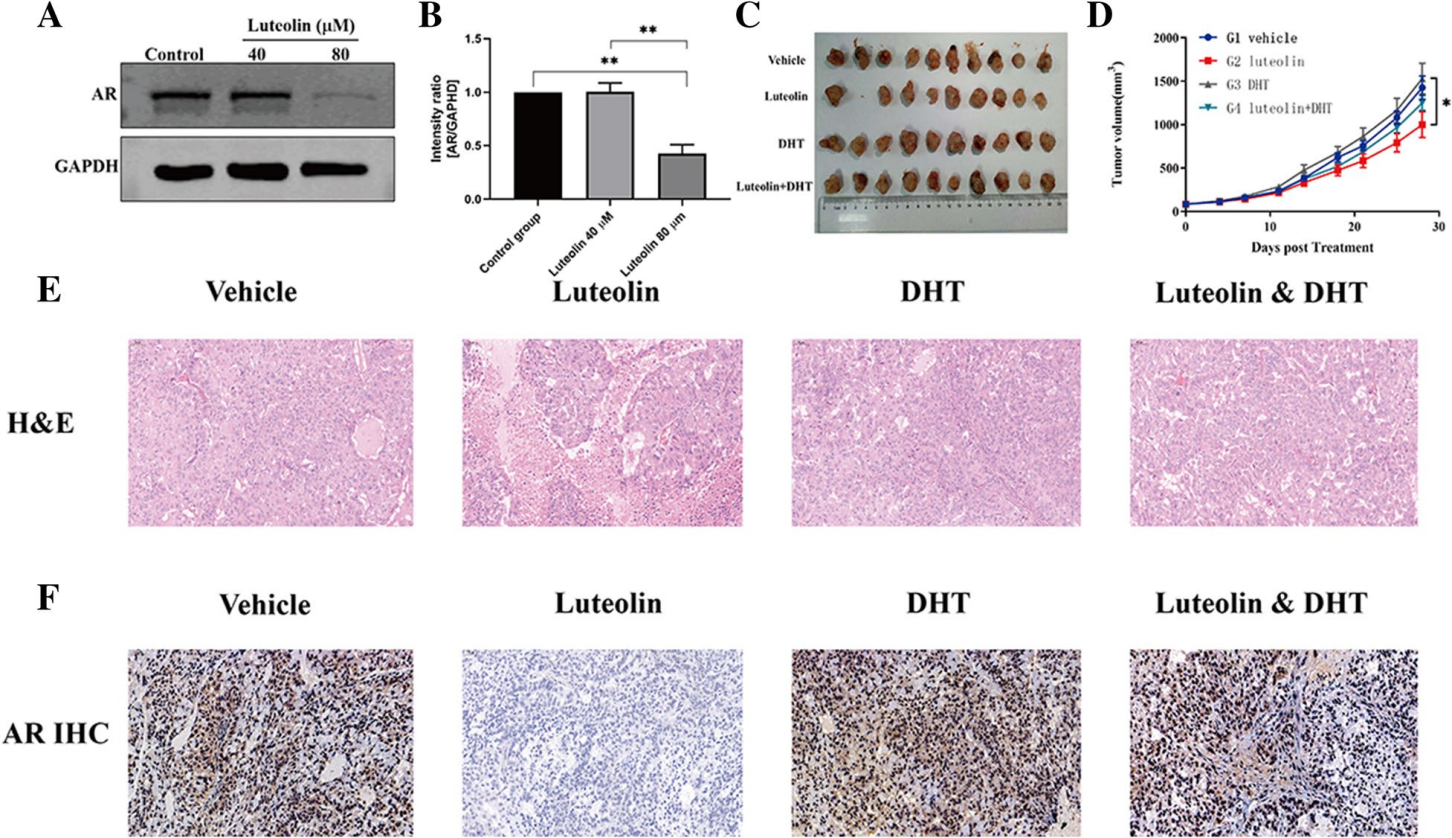
Luteolin inhibits AR receptor expression in A549. **A** Relative levels of AR in untreated A549 cells and luteolin treated with different concentrations detected by western blot analysis. **B** Column analysis of western blot analysis: *P < 0.05, **P < 0.01, ***P < 0.001, ****P < 0.0001. n = 3. **C** Xenograft tumor in each group of mice after treatment. **D** Xenograft tumor’s growth curve of mice (Mean ± SD, n = 10). **E** Hematoxylin eosin (HE) staining images showed nuclear atypia and high nuclear-cytoplasmic ratio. **F** Immunohistochemistry was performed on subcutaneous transplanted tumor of mice, AR in luteolin-treated group was consistent with in vitro

Western Blot showed that AR protein expression was different after treating A549 cells with different concentrations of luteolin for 48 h: When luteolin was 80 μM, AR protein expression was significantly decreased (Fig. 7A and B).

### Luteolin inhibits the growth of lung adenocarcinoma xenografts in vivo

To further evaluate the anti-tumor effects of luteolin, we engrafted A549 cells subcutaneously into nude mice. After the establishment of solid tumors, luteolin, DHT, luteolin + DHT or vehicle was administered every 2 days, respectively. We found that, compared with the control group, luteolin significantly inhibited the growth of A549 xenogeneic tumors at day 25 (*P* < 0.037) and 28 (*P* < 0.046) of administration, and compared with the DHT group, luteolin group also showed significant differences at day 25 (*P* < 0.046) and 28 (*P* < 0.035) of administration (Fig. 7C and D, Table 4). In addition, we examined the expression of AR in subcutaneous transplanted tumor of mice by immunohistochemistry. The level of AR positive staining was significantly reduced in the luteolin-treated group compared to the control group (Fig. 7E and F). Our results showed that luteolin suppresses tumor proliferation and AR expression.

**Table 4:** Statistical results of xenograft tumor growth in mice

	Group	PG-D25	PG-D28
		p值(TV)	p值(TV)
1	Vehicle	NA	NA
2	Luteolin	0.037	0.046
3	DHT	0.608	0.653
4	Luteolin + DHT	0.228	0.278
Note:	2vs3	0.046	0.035

## Discussion

TCM can regulate the pathogenic process of tumors so as to effectively and comprehensively treat the disease [5]. However, the composition of TCM is complex and can interact with multiple targets, so it is difficult to clarify the mechanism of action of TCM by traditional pharmacological methods [20]. Therefore, we sought to screen effective anti-tumor components among the TCM resources with limited toxic and side effects to find the balance between the benefits and risks of the efficacy and safety of TCM.

Network pharmacology was used to screen out the potential compound of YQG: luteolin. It not only has the characteristics of multi-target compounds, but also has better oral absorption and utilization efficiency in ADME prediction. Molecular docking results also showed that AR exhibited the highest LibDock score to luteolin. Luteolin had strong binding activity with AR. QSAR showed that luteolin and AR had high bioactivity values, which second only to AKT1 (AKT1). Since LibDock Score in molecular docking is a parameter for comprehensive evaluation of energy, geometry and chemical environment, and the significance of AR in lung cancer needs to be further confirmed.

Some studies have pointed out that luteolin has anti-tumor activity for many cancers [21]. In lung cancer, it can significantly inhibit the proliferation of KRAS mutant lung cancer, downregulate the expression of programmed death-ligand 1 induced by interferon-gamma, and reduce the migration of Lewis lung cancer cells in a CCL2-dependent manner [22–24]. Giulia D’Arrigo et al. [25] performed comparative computational docking simulations to predict the capability of six flavonoids (apigenin, genistein, luteolin, naringenin, quercetin, and resveratrol) to bind the AR, and its variant AR^T877A^ and membrane receptors for androgens. The results found all six flavonoids assumed an alternative orientation in AR^T877A^ orienting closer to helix12 (H12), and resembling the pose of known antagonists [26, 27]. In our study, combined with literature reports, identified the binding of luteolin to Thr 877 in AR as relatively significant by screening binding sites and phosphorylation sites. This hotspot T877A AR mutant loses the ability to differentiate agonists from antagonists [28]. Inactivation of the AR Thr 877 phosphorylation site may prevent luteolin from binding to the AR protein.

In order to further validate these findings, we firstly tested the effects of luteolin on the proliferation, migration and apoptosis of human lung cancer cells in vitro. The results showed that 80 μM luteolin significantly reduced proliferation and migration in A549 cells, however, the LDH test showed that its toxicity was weak. Flow cytometry for A549 cells showed no significant increase in apoptosis or necrosis after treatment with 80 μM luteolin alone. When 80 μM luteolin was used in combination with DDP (10 μg/mL), its effect on promoting apoptosis was better than that of DDP alone. Some studies believe that luteolin exerts pro-apoptotic effect on A549 lung adenocarcinoma cells [29, 30]. However, it has also been reported that luteolin has only a moderate anti-cancer effect on A549 cells [31]. In vascular smooth muscle cells, luteolin inhibited the proliferation and migration of A7r5 and HASMC cells, without affecting apoptosis [32]. Our results indicate that luteolin is a less toxic drug and can be used as adjunctive therapy in the treatment of non-small-cell lung cancer. These results were also similar to the reported results in some literature. Further apoptosis experiments are necessary to clarify the pro-apoptotic ability of luteolin on A549 cells, as well as its pro-apoptotic dose and duration of action.

Next, we used a plasmid construction approach to mutate AR (Thr-Ala877) so that this site exhibits allosteric inactivation and loss of phosphorylation before transfecting it into the A549 cell line. The results of CCK-8 and cell scratch assay showed that the proliferation and migration of A549 cells were inhibited to a certain extent by the treatment of 80 μM luteolin. The inhibitory effect of luteolin on the proliferation and migration of A549 transfected with AR wild plasmid and mutant plasmid was weaker than that of A549 transfected with un-transfected or empty vector. In particular, the inhibitory effect of luteolin was weaker after site mutation. The results of a western blot showed that AR expression was significantly inhibited by treatment with 80 μM luteolin. The transfection experiments revealed three possible problems: firstly, AR plays a role in promoting tumor proliferation and migration in A549. Secondly, luteolin may inhibit cell proliferation and migration mainly by binding to the phosphorylation site Thr (877) of the AR. Thirdly, luteolin could suppress the expression of AR protein and indirectly inhibit the proliferation of A549, which could explain the weak inhibitory effect of 80 μM luteolin on the proliferation of A549 even in AR mutant plasmid transfected A549.

In vivo experiment, luteolin administration was used to verify its anti-tumor effect in A549 xenograft mice. Although not all mouse transplanted tumors were effectively inhibited in this study, it was still observed that the anti-tumor effect of luteolin group began to appear at 25 days and 28 days compared with the control group and the DHT group. Additionally, immunohistochemical staining for AR expression in sections of mice transplanted tumors showed AR expression was decreased in luteolin group compared with other groups, which further validate the results of in vitro experiments.

Some studies have found that AR may play an important role in lung cancer biology, however, the role of AR signals in the progression of sex-unrelated cancers presents a complex situation and evidence remains contradictory [33–35]. In this study, using ligand binding and phosphorylation mutagenesis methods, we found that luteolin could inhibit AR protein expression and regulate tumor proliferation and migration by docking site Thr (877) of the AR.

In summary, we applied silico prediction to study the material basis and mechanism of YQG against lung cancer, and confirmed that luteolin can inhibit the proliferation and migration of lung cancer cells in vitro validation, and the mechanism of luteolin's action on lung cancer cells may be related to its docking at the phosphorylation site of THR 877 of the AR protein. This study also has some limitations. In this study, we selected the active compound with the highest score for docking with hub proteins for research through computer molecular docking, and we did not rule out the inhibitory effect of other active compounds on lung cancer cells. In addition, more studies are needed to further demonstrate if different AR-downstreams are modulated and if the AR-inhibition, in A549 or in the in vivo experiments induces the same effect induced by luteolin treatment, etc. More studies will be necessary in the future to further disentangle these questions.

This study preliminarily explored the mechanism of Chinese herbal compounds inhibiting lung cancer proliferation. These results seem to indicate that some flavonoids in Chinese herbal medicine inhibiting lung cancer are closely related to AR of lung cancer. Therefore, it seems that this direction of research is worthy of attention. They also provide some new directions for lung cancer therapy [19–22, 26, 31–33]

## Data Availability

All data generated during this study are included in this published article.
